# Prevalence and Incidence of Metabolic Syndrome and Its Components Among Waterpipe Users

**DOI:** 10.3389/ijph.2024.1607156

**Published:** 2024-07-11

**Authors:** Yasaman Sadeghi, Mahdokht Naghash, Hossein Poustchi, Saba Alvand, Abdullah Gandomkar, Hossein Molavi Vardanjani, Fatemeh Malekzadeh, Paolo Boffetta, Christian C. Abnet, Neal D. Freedman, Reza Malekzadeh, Arash Etemadi

**Affiliations:** ^1^ Digestive Oncology Research Center, Digestive Disease Research Institute, Tehran University of Medical Sciences, Tehran, Iran; ^2^ Liver and Pancreaticobilliary Research Center, Digestive Disease Research Institute, Tehran University of Medical Sciences, Tehran, Iran; ^3^ Non-Communicable Diseases Research Center, Shiraz University of Medical Sciences, Shiraz, Iran; ^4^ Medical Doctorate-Master of Public Health (MD-MPH) Program, School of Medicine, Research Center for Traditional Medicine and History of Medicine, Shiraz University of Medical Sciences, Shiraz, Iran; ^5^ Cancer Center, Stony Brook Medicine, Stony Brook, NY, United States; ^6^ Department of Medical and Surgical Sciences, University of Bologna, Bologna, Italy; ^7^ Metabolic Epidemiology Branch, Division of Cancer Epidemiology and Genetics, National Cancer Institute, National Institutes of Health (NIH), Bethesda, MD, United States

**Keywords:** waterpipe, metabolic syndrome, Iran, cross-sectional, prospective

## Abstract

**Objectives:**

To determine the associations between waterpipe use, duration, and intensity of use with prevalence and incidence of metabolic syndrome and its components (increased waist circumference, triglycerides, fasting glucose, blood pressure and decreased high-density lipoprotein cholesterol).

**Methods:**

We conducted cross-sectional and prospective analyses using data from the Pars Cohort Study in southern Iran, encompassing 9,264 participants at the baseline, and 5,002 randomly selected in a repeated follow-up. We used multivariate logistic regression models adjusted for age, sex, education, wealth score, physical activity and cigarette pack-years to report odds ratios (OR) and 95% confidence intervals (CI).

**Results:**

Among 9,264 participants, 3,119 (33.7%) had metabolic syndrome, and 3,482 (37.6%) had ever smoked waterpipe, with both more common in women than in men. In adjusted models, former waterpipe use was significantly associated with prevalence (OR = 1.43, 95% CI: 1.23–1.68) and incidence (OR = 1.57, 95% CI: 1.19–2.06) of the metabolic syndrome while current waterpipe use was not. Past use was associated with increased risk in all components of metabolic syndrome; current use was associated with increases in all except high blood glucose and hypertension. Past waterpipe users had higher waterpipe use intensity (before quitting) in comparison with current users (2.3 vs. 2.0 waterpipes per day, *p* < 0.01) and had started waterpipe smoking at a younger age (27.2 vs. 30.1 years, *p* < 0.01).

**Conclusion:**

Waterpipe use was associated with metabolic syndrome and its components, especially among former users potentially due to higher intensity and earlier initiation of use.

## Introduction

Waterpipe (also called hookah) is a way of smoking tobacco, which originated in the Middle East [1] but has become increasingly common worldwide, especially among the youth in Europe and the United States [1–3]. Waterpipe use has become a global epidemic with alarming trends, surpassing the prevalence of cigarette smoking in some countries [4]. Waterpipe use has been associated with increased overall and cancer-related mortality [5] and cardiovascular, pulmonary, and endocrine diseases, among others [2, 6]. It has also been associated with obesity [7, 8], hypertension [9, 10], increased fasting blood glucose [10–12], and dyslipidemia [10–12]. However, studies assessing the association of waterpipe use with the aggregation of these abnormalities (i.e., metabolic syndrome) are scarce.

The metabolic syndrome is a growing global health problem which can increase rates of all-cause mortality by up to 1.5 times [13], cardiovascular diseases by twofold [14–16], and type 2 diabetes mellitus by fivefold [14, 16]. The global prevalence of metabolic syndrome is reported at 12.5%–31.4% [17]. Cigarette smoking has been shown to be associated with metabolic syndrome and its components [11, 18–26]. The smoke inhaled during an average session of waterpipe use is equivalent to 39–172 cigarettes [2] and contains similar toxicants [2, 27, 28]. Consecutively, adverse health effects of waterpipe use may be similar to those of cigarette smoking [2, 6, 10, 29]. Even though waterpipe use has been associated with metabolic syndrome in a limited number of studies [9, 11, 30], studies with comprehensive lifetime assessment of exposure and those with a prospective design investigating the risk of metabolic syndrome incidence are lacking.

Pars Cohort Study (PCS) is a study conducted in Fars, a province in the south of Iran, which started in 2012 with a focus on non-communicable diseases [31]. There is a high prevalence of waterpipe use (37.5%) among PCS participants which is higher than the national average [32]. Detailed self-reported tobacco use histories were gathered using a questionnaire previously validated against cotinine levels [27]. These qualities along with the longitudinal nature of the study made PCS a unique platform to explore the association between waterpipe use and metabolic syndrome and its components. This is also the first study on these associations that uses the harmonized global definition of metabolic syndrome proposed in the 2009 Joint Interim Statement [14].

## Methods

Pars Cohort Study (PCS) started in 2012 in Valashahr District, a region in the South of Iran, with a population of over 40,000 inhabitants. They are mainly (95%) from Fars or Azari (Qashqai) ethnicities, which are the two major ethnic groups in the country, and live in the city of Valashahr and 93 villages. The sample of this study consisted of all eligible adults between 40 and 75. The response rate was 95% among residents who were invited from the city and nearby villages [31]. In a nested study, 5,002 participants were randomly selected for three repeated follow-up sessions with detailed laboratory data and drug histories in 2016, 2018, and 2021 [33]. Pars Cohort Study and its protocols were approved by ethical committees of Digestive Diseases Research Institute of Tehran University of Medical Sciences and Shiraz University of Medical Sciences.

At baseline, a general questionnaire was administered by trained interviewers which included self-reported personal, demographic and lifestyle characteristics such as education, questions addressing wealth level, physical activity, tobacco and alcohol use, plus medical history including disease and medication history. A composite wealth score was calculated based on house ownership, house size, and appliance ownership using multiple correspondence analysis as described before [34]. The wealth score was categorized into quartiles with 1 being the poorest to 4 being the wealthiest participants. Education was classified based on the years of formal schooling into four groups: “none,” “1–5 years,” “6–8 years” and “over 8 years.” Physical activity was determined using the Metabolic Equivalent of Task (MET, in minutes per week) and classified into quartiles.

We also collected a complete self-reported tobacco use profile at baseline, including the lifetime start and stop ages, daily consumption amounts, and frequency of use at each age using a detailed questionnaire (Supplementary Table S1). The questionnaire was validated in a previous study, showing 95.8% accuracy compared with urinary cotinine as the gold standard [27]. For cigarette and waterpipe use (each separately) ever users (vs. never users) were those who smoked cigarette/waterpipe at least once a week for 6 months and former users (vs. current users) were those who had stopped smoking cigarette/waterpipe at least 1 year before enrolling in this study.

Average waterpipe intensity (times per day) was calculated based on the individual’s average lifetime use and categorized into three groups: less than once a day, once or twice a day, and more than twice a day. Lifetime duration for waterpipe use was calculated as the number of years between the first use and their quitting time, or their current age (if they were still using), then categorized into tertiles: 10 years or fewer, 11–25 years, and more than 25 years.

We calculated cumulative cigarette use as pack-years [35, 36]by multiplying the duration of use by the average number of packs per day, across all periods of use. For example, someone who smoked 2 cigarette packs per day for 10 years, and someone who smoked 1 pack for 20 years both smoked a cumulative amount of 20 pack-years. We also calculated waterpipe cumulative use as waterpipe-years in the same way, and used the number of times per day instead of packs in the formula, as described before [5, 37]. Then we categorized the cumulative use into tertiles: 10 or fewer, 11–28, and more than 28 for cigarette pack-years; 10 or fewer, 11–48 and more than 48 for waterpipe-years.

Anthropologic indices (weight, height, waist and hip circumference) were measured and recorded by trained nurses and blood pressure was measured by a physician using a standard protocol [38]. BMI was calculated as weight (in kilograms) divided by squared height (in meters) and then classified based on WHO guidelines. Waist circumference was measured at umbilicus level, then divided into sex-specific quartiles. For each participant fasting blood glucose (FBG) and lipid profile were measured using commercial kits. The anthropologic measurements, blood pressure and laboratory measurements were repeated at the follow-up sessions along with updated drug histories.

We defined metabolic syndrome based on the 2009 Joint Interim Statement from AHA/NHLBI, World Heart Federation, International Atherosclerotic Society, and International Association for the Study of Obesity [14] which introduces a globally harmonized definition for metabolic syndrome. In this definition, metabolic syndrome is defined as the presence of 3 or more of the following criteria: 1. Elevated waist circumference (≥80 cm in women and ≥94 cm in men), 2. Elevated blood pressure (systolic blood pressure ≥130 mmHg and/or diastolic blood pressure ≥85 mmHg or antihypertensive drug treatment), 3. Elevated fasting glucose (fasting blood glucose ≥100 mg/dL or drug treatment of elevated glucose), 4. Reduced high-density lipoprotein cholesterol (HDL-C) (<50 mg/dL in women and <40 mg/dL in men or drug treatment for reduced HDL-C), 5. Elevated triglycerides (≥150 mg/dL or drug treatment for elevated triglycerides).

### Statistical Analysis

Descriptive data are presented as frequencies and percentages for categorical variables, and means and standard deviations for continuous variables. The chi-square test was used to compare categorical variables, and independent sample t-test was used for continuous variables. Our analysis consisted of a cross-sectional analysis using prevalent metabolic syndrome (for which we used the baseline data) and a prospective analysis to assess the risk of incident metabolic syndrome. Incident cases were defined as those who had a new diagnosis of metabolic syndrome during any of the three follow-up sessions and did not have metabolic syndrome at baseline. We used multiple logistic regression models to determine the adjusted odds ratios (ORs) and 95% confidence intervals (CIs). The main independent variables were waterpipe use (never, past, current), waterpipe use intensity (times per day), waterpipe use lifetime duration (in years), and cumulative waterpipe use (waterpipe-years) which were each used in separate models. Since past and current waterpipe users had different intensity and duration of use, two strategies were adopted: 1. Each correlate (intensity, duration and cumulative use) was stratified by past and current status, 2. The models including current and past waterpipe use were additionally adjusted for use intensity. Exclusive waterpipe use was also assessed in a separate model where cigarette smokers were excluded. The reference category in all these models (five models total for prevalence and five models total for incidence) was never waterpipe users. We adjusted the models for age, sex, education, wealth score, physical activity and cigarette pack-years. To make the models more parsimonious, we did not adjust our models for ethnicity and alcohol use as they did not have a significant effect on the estimates. A directed acyclic graph (DAG) was used to illustrate the relationships between variables and determine the confounding variables (Supplementary Figure S1). As both metabolic syndrome and waterpipe use varied significantly between men and women all analyses were also repeated stratified by sex.

The same adjusted models were used for the association of ever waterpipe use and each metabolic syndrome component (high blood pressure, high blood glucose, low HDL, high triglycerides, and high waist circumference as defined before). For prospective analysis, incident cases of each component were defined as new occurrences of the component during the follow-up together with the absence of the component at baseline.

We conducted two separate sensitivity analyses to investigate the possibility of reverse causation in which we excluded subjects with a self-reported history of chronic diseases (cardiovascular diseases, cerebrovascular accident, chronic obstructive pulmonary disease, chronic renal failure, jaundice and liver disease) once with and once without a history of cigarette smoking.

We used Stata software version 17 (StataCorp Inc., College Station, TX, United States) for statistical analyses. A two-sided *p*-value of <0.05 was considered statistically significant.

## Results

As seen in Table 1, among 9,264 participants, 4,276 (46.2%) were men and 4,988 (53.8%) were women, with a mean age of 52.6 (SD = 9.7). A total number of 3,119 (33.6%) fulfilled the criteria for the metabolic syndrome (MetS), including 965 men (22.6% of all men) and 2,154 women (43.2% of all women). The prevalence of the metabolic syndrome components from the most to least common at the baseline were as follows: high waist circumference (61.7%), high blood sugar (42.9%), high serum triglyceride (39.6%), high blood pressure (32.1%), and low serum HDL (19.7%). All components were significantly more common in women than in men (*p* < 0.01) (Supplementary Table S2).

**TABLE 1:** Baseline characteristics of the Pars cohort study population by metabolic syndrome and sex (Prevalence and Incidence of Metabolic Syndrome and Its Components among Waterpipe Users, Iran, 2024).

		Men (n = 4,276)		Women (n = 4,988)		Total (n = 9,264)	
		MetS+	MetS−	MetS+	MetS−	MetS+	MetS−
N (%)		965 (22.6)	3,311 (77.4)	2,154 (43.1)	2,834 (56.9)	3,119 (33.7)	6,145 (66.3)
Age (years): mean (SD)		53.4 (9.3)	52.5 (10.0)**	54.5 (9.7)	51.0 (9.0)**	54.1 (9.6)	51.8 (9.6)**
Ethnicity: n (%)	Qashqai	319 (33.0)	1,378 (41.6)**	771 (35.7)	1,128 (39.8)**	1,090 (34.9)	2,506 (40.7)**
	Other	646 (66.9)	1,933 (58.3)	1,383 (64.2)	1,706 (60.2)	2,029 (65.0)	3,639 (59.2)
Formal education: n (%)	None	263 (27.2)	1,077 (32.5)**	1,481 (68.7)	1,724 (60.8)**	1,744 (55.9)	2,801 (45.5)**
	=<5 years	252 (26.1)	936 (28.2)	586 (27.2)	957 (33.7)	838 (26.8)	1,893 (30.8)
	6–8 years	193 (20.0)	618 (18.6)	63 (2.9)	100 (3.5)	256 (8.2)	718 (11.6)
	>8 years	257 (26.6)	680 (20.5)	24 (1.1)	53 (1.8)	281 (9.0)	733 (11.9)
Wealth score quartiles: n (%)	1 (lowest)	378 (39.1)	851 (25.7)**	513 (23.8)	625 (22.0)*	891 (28.5)	1,476 (24.0)**
	2	243 (25.1)	870 (26.2)	535 (24.8)	641 (22.6)	778 (24.9)	1,511 (24.5)
	3	207 (21.4)	865 (26.1)	569 (26.4)	774 (27.3)	776 (24.8)	1,639 (26.6)
	4 (highest)	137 (14.2)	725 (21.9)	537 (24.9)	794 (28.0)	674 (21.6)	1,519 (24.7)
PA quartiles: n (%)	1 (lowest)	245 (25.8)	541 (16.7)**	743 (35.2)	738 (26.6)**	988 (32.3)	1,279 (21.3)**
	2	216 (22.8)	600 (18.5)	613 (29.0)	843 (30.4)	829 (27.1)	1,443 (24.0)
	3	198 (20.9)	785 (24.3)	537 (25.4)	769 (27.7)	735 (24.0)	1,554 (25.9)
	4 (highest)	288 (30.4)	1,301 (40.3)	218 (10.3)	417 (15.0)	506 (16.5)	1,718 (28.6)
Alcohol use: n (%)	Never	921 (95.4)	3,189 (96.3)	2,141 (99.4)	2,817 (99.4)	3,062 (98.1)	6,006 (97.7)
	Ever	44 (4.5)	122 (3.6)	13 (0.6)	17 (0.6)	57 (1.8)	139 (2.26)
Opium use: n (%)	Never	825 (85.5)	2,709 (81.8)	2,137 (99.2)	2,819 (99.4)	2,962 (95.0)	5,528 (90.0)
	Ever	140 (14.5)	602 (18.1)**	17 (0.7)	15 (0.5)	157 (5.0)	617 (10.0)**
Cigarette smoking: n (%)	Never	569 (58.9)	1,847 (55.7)**	2,136 (99.1)	2,815 (99.3)	2,705 (86.7)	4,662 (75.8)**
	Past	169 (17.5)	425 (12.8)	6 (0.3)	5 (0.2)	175 (5.6)	430 (7.0)
	Current	227 (23.5)	1,039 (31.3)**	12 (0.5)	14 (0.5)	239 (7.6)	1,053 (17.1)
Cigarette pack-years: n (%)	=<10	132 (13.6)	461 (13.9)	8 (0.3)	10 (0.3)	140 (4.4)	471 (7.6)**
	11–28	132 (13.6)	505 (15.5)	3 (0.1)	1 (0.0)	135 (4.3)	506 (8.2)
	>28	130 (13.4)	492 (14.8)	0	3 (0.1)	130 (4.1)	495 (8.0)
Waterpipe use: n (%)	Never	678 (70.2)	2,390 (72.1)**	1,096 (50.8)	1,618 (57.0)**	1,774 (56.8)	4,008 (65.2)**
	Past	158 (16.3)	409 (12.3)	499 (23.1)	459 (16.2)	657 (21.0)	868 (14.1)
	Current	129 (13.3)	512 (15.4)	559 (25.9)	757 (26.7)	688 (22.0)	1,269 (20.65)
Waterpipe-years: n (%)	=<10	105 (10.8)	285 (8.6)	358 (16.6)	451 (15.9)**	463 (14.8)	736 (11.9)**
	11–48	75 (7.7)	270 (8.1)	363 (16.8)	415 (14.6)	438 (14.0)	685 (11.1)
	>48	107 (11.0)	366 (11.0)	337 (15.6)	350 (12.3)	444 (14.2)	716 (11.6)
BMI (kg/m^2^): n (%)	<18	6 (0.6)	269 (8.1)**	5 (0.2)	111 (3.9)**	11 (0.3)	380 (6.1)**
	18–24.9	176 (18.2)	1997 (60.3)	416 (19.3)	1,137 (40.1)	592 (18.9)	3,134 (51.0)
	25–29.9	549 (56.8)	880 (26.5)	984 (45.6)	1,037 (36.5)	1,533 (49.1)	1,917 (31.2)
	≥30	234 (24.2)	165 (4.9)	749 (34.7)	549 (19.3)	983 (31.5)	714 (11.6)
WC (cm): mean (SD)		99.2 (9.7)	85.8 (10.4)**	98.0 (10.6)	89.3 (12.7)**	98.4 (10.4)	87.4 (11.6)**
SBP (mmHg): mean (SD)		121.3 (18.7)	108.6 (16.6)**	120.0 (21.3)	106.5 (16.5)**	120.4 (20.6)	107.6 (16.6)**
DBP (mmHg): mean (SD)		79.7 (11.3)	71.9 (11.3)**	77.2 (12.7)	70.1 (10.5)**	78.0 (12.3)	71.1 (11.0)**
FBS (mg/dL): mean (SD)		119.4 (47.3)	98.4 (23.2)**	121.3 (52.2)	97.3 (23.7)**	120.6 (50.8)	97.9 (23.5)**
HDL (mg/dL): mean (SD)		47.9 (10.8)	56.1 (11.4)**	55.9 (12.8)	64.1 (12.3)**	53.4 (12.8)	59.8 (12.5)**
TG (mg/dL): mean (SD)		241.3 (173.6)	129.1 (73.0)**	206.9 (117.3)	116.4 (57.0)**	217.5 (138.1)	123.3 (66.4)**
LDL (mg/dL): mean (SD)		103.7 (36.0)	101.9 (31.0)	113.5 (37.9)	109.5 (32.5)**	110.5 (37.6)	105.4 (31.9)**

MetS, metabolic syndrome; SD, standard deviation; PA, physical activity; BMI, body mass index; WC, waist circumference; SBP, systolic blood pressure; DBP, diastolic blood pressure; FBS, fasting blood sugar; HDL, high-density lipoprotein; TG, triglyceride; LDL, low-density lipoprotein.

*p < 0.05 **p < *0.01*.

The metabolic syndrome had a positive significant association with age, it was more common among ethnicities other than Qashqai (the most common ethnic group in this population), and it showed an inverse significant relationship with education, wealth score and physical activity in both men and women. Prevalent metabolic syndrome was inversely associated with ever opium use and cigarette smoking. We did not find any associations between the metabolic syndrome and alcohol use (Table 1).

Almost all the tobacco used in this population was either in the form of cigarettes or waterpipe, except for 13 people who also chewed tobacco. A total number of 3,482 (37.6%) people reported ever waterpipe use (past and current), including 1,208 men (28.3% of all men) and 2,274 women (45.6% of all women). Waterpipe use was significantly more common in women (*p* < 0.01) (Supplementary Table S2). Cigarette smoking was rare among women (past: 0.2%, current: 0.5%) compared to men (past: 13.8%, current: 29.6%). Waterpipe users were significantly older, wealthier and less educated than non-users. Waterpipe use was more common among past cigarette smokers compared with never and current cigarette smokers and was inversely associated with cigarette pack-years (Supplementary Table S3). Past waterpipe users had higher waterpipe use intensity (before quitting) in comparison with current users (2.3 vs. 2.0 waterpipes per day, *p* < 0.01) and had started waterpipe smoking at a younger age (27.2 vs. 30.1 years, *p* < 0.01).

The findings of the logistic regression analysis for prevalent and incident metabolic syndrome are presented in Table 2, consisting of the total population and sex-stratified estimates. Below, the total population estimates are explained, with sex-stratified estimates detailed if discordant.

**TABLE 2:** The association of correlates of waterpipe use with prevalence and incidence of metabolic syndrome in adjusted logistic regression models (Prevalence and Incidence of Metabolic Syndrome and Its Components among Waterpipe Users, Iran, 2024).

		Prevalent metabolic syndrome				Incident metabolic syndrome			
		Number	Men OR (95%CI)^a^	Women OR (95%CI)^a^	Total OR (95%CI)^b^	Number	Men OR (95% CI)^a^	Women OR (95% CI)^a^	Total OR (95% CI)^b^
WP use ^c^	Never	5,782	Reference	Reference	Reference	2,939	Reference	Reference	Reference
	Past	1,525	1.53** (1.16–2.02)	1.37** (1.13–1.66)	1.43** (1.23–1.68)	1,001	1.71** (1.16–2.52)	1.44 (0.97–2.12)	1.57** (1.19–2.06)
	Current	1,957	1.01 (0.77–1.33)	1.16 (0.98–1.38)	1.14 (0.99–1.31)	1,062	1.05 (0.73–1.52)	1.20 (0.85–1.70)	1.14 (0.88–1.46)
Exclusive WP use ^c^ ^,^ ^d^	Never	4,418	Reference	Reference	Reference	2,226	Reference	Reference	Reference
	Past	1,204	1.70* (1.13–2.57)	1.34** (1.11–1.63)	1.41** (1.18–1.67)	805	1.53 (0.89–2.63)	1.44 (0.97–2.13)	1.45* (1.05–2.00)
	Current	1,725	1.02 (0.71–1.45)	1.15 (0.97–1.36)	1.13 (0.97–1.32)	935	0.84 (0.53–1.34)	1.21 (0.85–1.71)	1.07 (0.81–1.41)
Waterpipe years	Never smoker	5,782	Reference	Reference	Reference	2,939	Reference	Reference	Reference
	Past & wp-year ≤10	571	1.52* (1.08–2.13)	1.31* (1.05–1.65)	1.37* (1.14–1.65)	347	1.16 (0.71–1.92)	1.08 (0.68–1.70)	1.11 (0.79–1.55)
	Past & wp-year 11–48	499	1.36 (0.95–1.94)	1.34* (1.05–1.70)	1.36* (1.11–1.65)	322	1.23 (0.76–1.98)	1.13 (0.69–1.87)	1.18 (0.84–1.67)
	Past & wp-year >48	455	1.24 (0.88–1.77)	1.28 (0.97–1.68)	1.26** (1.02–1.55)	332	1.48 (0.95–2.32)	1.22 (0.72–2.06)	1.37 (0.97–1.92)
	Current & wp-year ≤10	628	1.21 (0.86–1.72)	1.14 (0.92–1.41)	1.16 (0.96–1.39)	241	1.41 (0.82–2.41)	1.02 (0.63–1.66)	1.19 (0.83–1.71)
	Current & wp-year 11–48	624	0.68 (0.44–1.06)	1.17 (0.95–1.45)	1.06 (0.88–1.27)	333	0.66 (0.38–1.13)	1.18 (0.76–1.82)	0.95 (0.69–1.31)
	Current & wp-year >48	705	0.89 (0.64–1.23)	1.05 (0.85–1.30)	1.00 (0.84–1.20)	488	0.71 (0.49–1.03)	0.83 (0.56–1.21)	0.77 (0.59–1.00)
WP duration	Never smoker	5,782	Reference	Reference	Reference	2,939	Reference	Reference	Reference
	Past & <10 years	718	1.39** (1.03–1.88)	1.33* (1.08–1.64)	1.36* (1.14–1.61)	441	1.20 (0.78–1.85)	1.05 (0.69–1.59)	1.12 (0.83–1.51)
	Past & 11–25 years	460	1.22 (0.84–1.76)	1.48* (1.15–1.90)	1.38* (1.12–1.69)	306	1.20 (0.75–1.90)	1.09 (0.65–1.84)	1.15 (0.81–1.63)
	Past & >25 years	347	1.55** (1.04–2.32)	1.08 (0.80–1.45)	1.23 (0.97–1.57)	254	1.67 (0.96–2.89)	1.37 (0.77–2.45)	1.52* (1.02–2.26)
	Current & <10 years	617	1.08 (0.76–1.53)	1.22 (0.98–1.51)	1.17 (0.98–1.41)	250	1.13 (0.68–1.89)	0.77 (0.48–1.22)	0.91 (0.65–1.29)
	Current & 11–25 years	579	0.82 (0.54–1.23)	1.06 (0.85–1.33)	1.00 (0.82–1.21)	246	0.80 (0.44–1.43)	1.76* (1.03–3.00)	1.25 (0.86–1.81)
	Current & >25 years	760	0.89 (0.64–1.25)	1.10 (0.90–1.34)	1.05 (0.89–1.24)	565	0.71 (0.49–1.03)	0.84 (0.58–1.20)	0.79 (0.61–1.01)
WP intensity	Never smoker	5,782	Reference	Reference	Reference	2,939	Reference	Reference	Reference
	Past & <1 times/day	465	1.58** (1.04–2.41)	1.36* (1.08–1.72)	1.41* (1.15–1.73)	277	1.54 (0.82–2.89)	1.15 (0.69–1.91)	1.29 (0.87–1.93)
	Past & 1–2 times/day	491	1.53* (1.08–2.16)	1.24 (0.97–1.58)	1.33* (1.09–1.63)	339	1.38 (0.85–2.24)	1.34 (0.82–2.20)	1.35 (0.96–1.90)
	Past & >2 times/day	560	1.20 (0.89–1.64)	1.34** (1.03–1.72)	1.28* (1.06–1.55)	382	1.20 (0.81–1.77)	0.94 (0.58–1.51)	1.09 (0.81–1.48)
	Current & <1 times/day	749	1.01 (0.70–1.46)	1.12 (0.93–1.36)	1.09 (0.93–1.29)	338	0.92 (0.55–1.53)	1.28 (0.83–1.97)	1.14 (0.83–1.58)
	Current & 1–2 times/day	554	1.04 (0.69–1.56)	1.22 (0.98–1.51)	1.19 (0.98–1.44)	331	0.97 (0.58–1.65)	0.77 (0.51–1.17)	0.84 (0.60–1.16)
	Current & >2 times/day	636	0.80 (0.58–1.10)	1.01 (0.80–1.28)	0.94 (0.78–1.13)	384	0.70 (0.48–1.03)	0.91 (0.59–1.41)	0.80 (0.60–1.05)

OR, odds ratio; CI, confidence interval; WP, waterpipe.

*p < 0.05 **p < 0.01.

^a^ Logistic regression models adjusted for age, education, wealth score, physical activity and cigarettes pack-year.

^b^ Logistic regression models adjusted for age, sex, education, wealth score, physical activity and cigarettes pack-year.

^c^ These models were also adjusted for intensity of waterpipe use.

^d^ Exclusive waterpipe use state was calculated after excluding cigarette smokers from the model.

### Past and Current Waterpipe Use

There were significant positive associations between past waterpipe use and prevalent metabolic syndrome (OR = 1.43, 95% CI: 1.23–1.68) and incident metabolic syndrome (OR = 1.57, 95% CI: 1.19–2.06), although the association with incidence was not significant among women. After the exclusion of cigarette smokers, the association of past use with metabolic syndrome remained significant for prevalence (OR = 1.41, 95% CI: 1.18–1.67), but the association with incidence was only significant in the total population (OR = 1.45, 95% CI: 1.05–2.00) and not the sex strata. When we excluded individuals with a history of chronic diseases as a sensitivity analysis (Supplementary Table S4), the association between past waterpipe smoking and prevalent metabolic syndrome weakened (OR = 1.33, 95% CI: 1.11–1.60).

There were no significant associations between current waterpipe use and the metabolic syndrome. In the sensitivity analysis excluding those with a history of chronic diseases (Supplementary Table S4), current use showed a statistically significant association (OR = 1.21; 95% CI: 1.04–1.42) albeit non-significant among men.

### Cumulative Waterpipe Use

There were significant associations between all levels of cumulative waterpipe use and prevalence of metabolic syndrome among past waterpipe users. In women, only the association for former users with above 48 waterpipe-years was not statistically significant, while in men only the association between waterpipe-years ≤10 and the prevalent metabolic syndrome reached statistical significance. Associations between waterpipe-years and incident metabolic syndrome were similar but insignificant. No significant association was found between waterpipe cumulative use and metabolic syndrome among current users.

### Duration of Waterpipe Use

Duration of use was significantly associated with prevalent metabolic syndrome among past users but not current users. Less than 10 years and 11–25 years of use among past waterpipe users were associated with prevalent metabolic syndrome with odds ratios of 1.36 (95% CI: 1.14–0.61) and 1.38 (95% CI: 1.12–1.69), respectively with the latter not statistically significant among men. Over 25 years of duration of use among former waterpipe users was significantly associated with metabolic syndrome incidence only in the total population (OR = 1.52, 95% CI: 1.02–2.26) but not among men and women separately.

### Intensity of Waterpipe Use

All categories of intensity were associated with prevalent metabolic syndrome among past users but not current users. Daily intensities of less than 1 session, 1–2 times, and above 2 times were associated with prevalent metabolic syndrome with odds ratios of 1.41 (95% CI: 1.15–1.73), 1.33 (95% CI: 1.09–1.63), and 1.28 (95% CI: 1.06–1.55). Sex-stratified results of former users were similar to that of the total population except for an intensity of >2 times per day among men and 1–2 times per day among women. While no significant association was found between intensity of use and metabolic syndrome incidence, the associations were generally in accordance with those of the prevalence assessment.

As Table 3 shows, in adjusted models, past waterpipe use was significantly associated with increased prevalence of high waist circumferences (OR = 1.81, 95% CI: 1.49–2.19), prevalence and incidence of low HDL (OR = 1.25, 95% CI: 1.05–1.49 and OR = 1.34, 95% CI: 1.07–1.69, respectively), the prevalence and incidence of high triglycerides (OR = 1.17, 95% CI: 1.01–1.36 and OR = 1.39, 95% CI: 1.08–1.78, respectively), prevalence and incidence of increased blood glucose (OR = 1.17, 95% CI: 1.01–1.36 and OR = 1.69, 95% CI: 1.29–2.21), and prevalence of high blood pressure (OR = 1.44, 95% CI: 1.23–1.69). Current waterpipe use was associated with increased prevalence of high waist circumferences (OR = 1.42, 95% CI: 1.20–1.69), prevalence and incidence of low HDL (OR = 1.20, 95% CI: 1.02–1.40 and OR = 1.32, 95% CI: 1.06–1.64, respectively), and incidence of high serum triglycerides (OR = 1.39, 95% CI: 1.09–1.77). Current use was also associated with a reduction in the prevalence of high blood pressure (OR = 0.85, 95% CI: 0.73–0.98).

**TABLE 3:** The association of past and current waterpipe use with components of metabolic syndrome in adjusted logistic regression models for prevalent and incident cases of metabolic syndrome by sex (Prevalence and Incidence of Metabolic Syndrome and Its Components among Waterpipe Users, Iran, 2024).

		Prevalent metabolic syndrome			Incident metabolic syndrome		
	Waterpipe use^a^	Men OR (95% CI)^b^	Women OR (95% CI)^b^	Total OR (95% CI)^c^	Men OR (95% CI)^b^	Women OR (95% CI)^b^	Total OR (95% CI)^c^
High WC	Past	1.63** (1.26–2.12)	2.02** (1.51–2.69)	1.81** (1.49–2.19)	1.52 (1.00–2.31)	0.95 (0.46–1.99)	1.35 (0.94–1.94)
	Current	1.21 (0.95–1.54)	1.65** (1.30–2.10)	1.42** (1.20–1.69)	1.13 (0.75–1.71)	0.62 (0.31–1.23)	0.98 (0.69–1.40)
High blood pressure	Past	1.90** (1.45–2.50)	1.22 (1.00–1.49)	1.44** (1.23–1.69)	1.29 (0.87–1.90)	0.93 (0.66–1.31)	1.05 (0.81–1.35)
	Current	0.95 (0.73–1.25)	0.79* (0.65–0.95)	0.85* (0.73–0.98)	0.87 (0.61–1.23)	0.82 (0.61–1.09)	0.83 (0.67–1.04)
High blood sugar	Past	1.06 (0.84–1.35)	1.21 (1.00–1.47)	1.17* (1.01–1.36)	1.59* (1.02–2.49)	1.74** (1.24–2.46)	1.69** (1.29–2.21)
	Current	0.82 (0.66–1.03)	1.02 (0.86–1.21)	0.94 (0.82–1.08)	1.00 (0.65–1.54)	1.08 (0.79–1.48)	1.05 (0.81–1.35)
Low HDL	Past	1.22 (0.88–1.69)	1.25* (1.02–1.55)	1.25* (1.05–1.49)	1.42* (1.02–1.98)	1.28 (0.92–1.76)	1.34* (1.07–1.69)
	Current	0.82 (0.58–1.14)	1.30** (1.08–1.57)	1.20* (1.02–1.40)	1.18 (0.85–1.64)	1.40* (1.03–1.90)	1.32* (1.06–1.64)
High TG	Past	1.20 (0.94–1.54)	1.12 (0.93–1.36)	1.17* (1.01–1.36)	1.93** (1.27–2.91)	1.13 (0.82–1.55)	1.39* (1.08–1.78)
	Current	1.04 (0.83–1.31)	1.16 (0.98–1.38)	1.13 (0.99–1.30)	1.54* (1.02–2.32)	1.31 (0.96–1.78)	1.39** (1.09–1.77)

OR, odds ratio; CI, confidence interval; WC, waist circumference; HDL, high-density lipoprotein; TG, triglyceride.

*p < 0.05 **p < 0.01.

^a^ The reference category in all models was never waterpipe use.

^b^ Logistic regression models adjusted for age, education, wealth score, physical activity, intensity of waterpipe use, and cigarettes pack-year.

^c^ Logistic regression models adjusted for age, sex, education, wealth score, physical activity, intensity of waterpipe use, and cigarettes pack-year.

## Discussion

In this population of Southern Iran, both the metabolic syndrome and waterpipe smoking were more common among women. Past waterpipe smokers had higher intensity of use and had started smoking at lower ages compared with current waterpipe smokers. We found a statistically significant association between waterpipe use, its intensity and duration and both prevalent and incident metabolic syndrome among former users. When we excluded individuals with a history of chronic diseases, these associations weakened. Former waterpipe use compared to never use is associated with an increased likelihood of all metabolic syndrome components and with the risk of incidence for increased blood glucose, increased triglyceride, and low HDL. Current waterpipe use was associated with an increase in the incidence of high triglyceride and low HDL, an increase in the prevalence of high waist circumference and low HDL, along with a decrease in prevalence of hypertension.

Women were more likely to use waterpipe in our population, while in many countries around the world the rate of use is higher among men [39]. Studies have shown that women in West Asia and North Africa prefer using waterpipe to cigarettes due to social acceptance [40, 41]. About one-third of our participants had the metabolic syndrome (33.6%). It is not easy to determine the global prevalence of the metabolic syndrome due to different definitions used for it [42], but in the year 2022, it was estimated to be 31.4% among the general adult population based on the Joint Interim Statement used in our study [17]. According to our results, metabolic syndrome was more common among women, which was consistent with previous studies from Iran [43, 44]. In our population, women were physically less active and had higher rates of central obesity compared to men (85.5% in women vs. 34.1% in men).

Previous studies on cigarette smoking have shown a positive association with the metabolic syndrome [20, 21, 23–26] and this association was dose-dependent in some studies [18, 22]. This association was also seen in both direct tobacco smoking and exposure to second-hand tobacco smoke [45]. According to our results, past waterpipe use was associated with the metabolic syndrome, but current waterpipe use was not. This was consistent with a previous study conducted by Soltani et al, who investigated the relationship between waterpipe smoking and the metabolic syndrome in individuals 60 or older, and found that former waterpipe users had higher rates of the metabolic syndrome in comparison with never and current users [30]. Another study by Soflaei et al showed that waterpipe use was associated with increased prevalence of metabolic syndrome while cigarette smoking was not [11]. Other studies have also showed significant associations between current waterpipe use and the metabolic syndrome [29]. The reason for these differences is not clear, but past waterpipe smokers in our study had a significantly higher intensity of waterpipe use and had started smoking waterpipe at younger ages than current users which may have led the associations to be stronger in past users. In all likelihood, heavier use and earlier start may have led to adverse health reactions and more doctors’ visits recommending quitting, as also shown in a previously conducted study on quitting patterns among cigarette smokers above the age of 44 [46]. This potential reverse causation effect was also observed with the weakening of the associations among past waterpipe users with the exclusion of individuals with a history of chronic diseases.

Cigarette smoking has been shown to affect body fat distribution. Cigarette smokers have higher rates of central obesity despite lower BMI in comparison with non-smokers [47]. Past smokers have higher rates of increased waist circumference and lowered HDL in comparison with never smokers [48]. The effect of quitting smoking on weight gain and has been shown in several studies [26] and can explain the stronger associations between past waterpipe use, compared with current use, and both waist circumference and metabolic syndrome. In our study, both past and current waterpipe users had higher waist circumferences compared with non-users, as well as higher levels of serum triglycerides and lower HDL. Soltani et al found associations between past waterpipe use with central obesity and current waterpipe use with decreased HDL levels [30]. Soflaei et al showed an association between current waterpipe use and both increased waist circumference and decreased HDL levels [11]. The positive relationship between waterpipe use and obesity has also been shown in other studies [9, 49]. Another study showed that daily waterpipe users had a three-fold higher risk for obesity in comparison with never-users [50]. Increased risk of metabolic syndrome among tobacco users may also be due to unhealthy lifestyle and lack of physical activity, but our analyses were adjusted for physical activity.

Previous results on the relationship between waterpipe use and blood pressure have been controversial. Some studies have shown an association between waterpipe use and high blood pressure [9, 51, 52]. On the other hand, there have been reports of reduction in heart rate and blood pressure in waterpipe users [30, 53, 54]. We showed that while past waterpipe users had higher blood pressure than non-users, current waterpipe users had lower blood pressure. A study conducted on awareness and risk factors of hypertension in Iran showed that compared to non-smokers, current but not past smokers had lower blood pressures, and past smokers had higher awareness of their health condition [55], so they might have quit smoking because of the health consequences. Furthermore, another study on preventive lifestyle behaviours in hypertension conducted in Canada proposed a positive association between perceiving hypertension as a health hazard due to smoking cigarettes and quitting smoking to control blood pressure [56].

We studied a population with relatively high rates of both waterpipe smoking and the metabolic syndrome. We used longitudinal data for metabolic syndrome incidence and a validated self-reported comprehensive tobacco use history at baseline for exposure assessment.

### Limitations

While we found significant associations between waterpipe use and metabolic syndrome, the associations with past waterpipe use may be partly due to reverse causation or residual confounding. Though national surveys confirm the low proportion of alcohol use in this population [57], we cannot completely rule out the likelihood of underreporting due to religious beliefs. Tobacco use histories were not based on objective methods, however, our self-report questionnaire has been validated previously against cotinine values.

### Conclusion

We found significant associations between waterpipe smoking and the metabolic syndrome components, as well as the syndrome itself. Given the alarming increase in the popularity of waterpipe use, our findings build on the diverse adverse health impacts of this method of tobacco use, highlighting the need for preventive health strategies. Further experimental research on the potential mechanism of the effects of waterpipe use on metabolic syndrome and larger prospective studies are warranted to clarify the observed associations.
