# Supplementary material for: Prevalence and Incidence of Metabolic Syndrome and Its Components Among Waterpipe Users
Source: Int J Public Health. 2024 Jul 11;69:1607156. doi: 10.3389/ijph.2024.1607156 (PMC11269743; doi:10.3389/ijph.2024.1607156)

**Supplementary Table S1 – Tobacco use questionnaire items and their corresponding variables used for analysis (Prevalence and Incidence of Metabolic Syndrome and Its Components among Waterpipe Users, Iran, 2024)**

8.1 Have you ever smoked cigarettes regularly (at least weekly over a 6 month period)?

(if No, go to 8.4)

(1) Yes (2) No |__|

8.2 Do you still smoke cigarettes now? (1) Yes (2) No (8) Never smoked |__|

8.3 List cigarette consumption beginning with when you started to smoke regularly:

| From age | To  age | *Type | Number per day | Days per week |
| --- | --- | --- | --- | --- |
| \|__\|\|__\| | \|__\|\|__\| | \|__\| | \|__\|\|__\| | \|__\| |
| \|__\|\|__\| | \|__\|\|__\| | \|__\| | \|__\|\|__\| | \|__\| |
| \|__\|\|__\| | \|__\|\|__\| | \|__\| | \|__\|\|__\| | \|__\| |
| \|__\|\|__\| | \|__\|\|__\| | \|__\| | \|__\|\|__\| | \|__\| |
| \|__\|\|__\| | \|__\|\|__\| | \|__\| | \|__\|\|__\| | \|__\| |

8.4 Have you ever used other types of tobacco regularly? (at least weekly over a 6 month period)

(1) Yes (2) No |__|

8.4.1 If yes, list consumption:

| Product | Ever used  (1)Yes | If ever used | | | |
| --- | --- | --- | --- | --- | --- |
|  |  | Age when first smoked | Age when last smoked | Usual number  per day | Days/week |
| Nass | \|__\|  \|__\|  \|__\| | \|__\|\|__\|  \|__\|\|__\|  \|__\|\|__\| | \|__\|\|__\|  \|__\|\|__\|  \|__\|\|__\| | \|__\|\|__\|  \|__\|\|__\|  \|__\|\|__\| | \|__\|  \|__\|  \|__\| |
| Calumet (traditional pipe) | \|__\|  \|__\|  \|__\| | \|__\|\|__\|  \|__\|\|__\|  \|__\|\|__\| | \|__\|\|__\|  \|__\|\|__\|  \|__\|\|__\| | \|__\|\|__\|  \|__\|\|__\|  \|__\|\|__\| | \|__\|  \|__\|  \|__\| |
| Pipe | \|__\|  \|__\|  \|__\| | \|__\|\|__\|  \|__\|\|__\|  \|__\|\|__\| | \|__\|\|__\|  \|__\|\|__\|  \|__\|\|__\| | \|__\|\|__\|  \|__\|\|__\|  \|__\|\|__\| | \|__\|  \|__\|  \|__\| |
| Hookah | \|__\|  \|__\|  \|__\| | \|__\|\|__\|  \|__\|\|__\|  \|__\|\|__\| | \|__\|\|__\|  \|__\|\|__\|  \|__\|\|__\| | \|__\|\|__\|  \|__\|\|__\|  \|__\|\|__\| | \|__\|  \|__\|  \|__\| |

**Supplementary Table S2. Waterpipe, metabolic syndrome and its components among the Pars cohort study baseline participants, by sex (Prevalence and Incidence of Metabolic Syndrome and Its Components among Waterpipe Users, Iran, 2024)**

|  | Men  (n=4276) | Women  (n=4988) | Total  (n=9264) |
| --- | --- | --- | --- |
| Metabolic syndrome: n (%) | 965 (22.6) | 2154 (43.2)** | 3119 (33.6) |
| High WC: n (%) | 1457 (34.1) | 4263 (85.5)** | 5720 (61.7) |
| High blood pressure: n (%) | 1175 (27.4) | 1803 (36.1)** | 2978 (32.1) |
| High fasting glucose: n (%) | 1760 (41.2) | 2221 (44.5)** | 3981 (43.0) |
| Low HDL: n (%) | 544 (12.8) | 1283 (25.8)** | 1827 (19.8) |
| High TG: n (%) | 1631 (38.1) | 2038 (40.9)** | 3669 (39.6) |
| WP ever use: n (%) | 1208 (28.2) | 2274 (45.6)** | 3482 (37.6) |
| WC: waist circumference, HDL: high-density lipoprotein, TG: triglycerides, WP: waterpipe  ***p<0.01* | | | |

|  |  | **Men**  (n=4276) | | **Women**  (n=4988) | | **Total**  (n=9264) | |
| --- | --- | --- | --- | --- | --- | --- | --- |
|  |  | **waterpipe +**  (1208, 28.2) | **waterpipe -**  (3068, 71.7) | **waterpipe +**  (2274, 45.5) | **waterpipe -**  (2714, 54.4) | **waterpipe +**  (3482, 37.5) | **waterpipe -**  (5782, 62.4) |
| Age (years) |  | 55.2 (10.7) | 51.7 (9.3)** | 54.0 (9.6) | 51.2 (9.1)** | 54.4 (10.0) | 51.5 (9.2)** |
| Ethnicity: n (%) | Qashqai | 471 (27.7) | 1226 (72.2) | 852 (44.8) | 1047 (55.1) | 1323 (36.7) | 2273 (63.2) |
|  | Other | 737 (28.5) | 1842 (71.4) | 1422 (46.0) | 1667 (53.9) | 2159 (38.0) | 3509 (61.9) |
| Education: n (%) | None | 512 (38.2) | 828 (61.7)** | 1622 (50.6) | 1583 (49.3)** | 2134 (46.9) | 2411 (53.0)** |
|  | 5 years>= | 335 (28.2) | 853 (71.8) | 581 (37.6) | 962 (62.3) | 916 (33.5) | 1815 (66.4) |
|  | 6-8 years | 187 (23.0) | 624 (76.9) | 50 (30.6) | 113 (69.3) | 237 (24.3) | 737 (75.6) |
|  | High school | 134 (19.8) | 540 (80.1) | 16 (27.1) | 43 (72.8) | 150 (20.4) | 583 (79.5) |
|  | University | 40 (15.2) | 223 (84.7) | 5 (27.7) | 13 (72.2) | 45 (16.0) | 236 (83.9) |
| Wealth score quartiles: n (%) | 1 (lowest) | 288 (23.4) | 941 (76.5)** | 443 (38.9) | 695 (61.0)** | 731 (30.8) | 1636 (69.1)** |
|  | 2 | 304 (27.3) | 809 (72.6) | 537 (45.6) | 639 (54.3) | 841 (36.7) | 1448 (63.2) |
|  | 3 | 318 (29.6) | 754 (70.3) | 617 (45.9) | 726 (54.0) | 935 (38.7) | 1480 (61.2) |
|  | 4 (highest) | 298 (34.5) | 564 (65.4) | 677 (50.8) | 654 (49.1) | 975 (44.4) | 1218 (55.5) |
| Physical activity quartiles: n (%) | 1 (lowest) | 236 (30.0) | 550 (69.9) | 669 (45.1) | 812 (54.8)** | 905 (39.9) | 1362 (60.0)** |
|  | 2 | 241 (29.5) | 575 (70.4) | 654 (44.9) | 802 (55.0) | 895 (39.3) | 1377 (60.6) |
|  | 3 | 273 (27.7) | 710 (72.2) | 596 (45.6) | 710 (54.3) | 869 (37.9) | 1420 (62.0) |
|  | 4 (highest) | 427 (26.8) | 1162 (73.1) | 312 (49.1) | 323 (50.8) | 739 (33.2) | 1485 (66.7) |
| Alcohol use: n (%) |  | 55 (33.1) | 111 (66.8) | 21 (70.0) | 9 (30.0) | 76 (38.7) | 120 (61.2) |
| Opium use: n (%) |  | 218 (29.3) | 524 (70.6) | 17 (53.1) | 15 (46.8) | 235 (30.3) | 539 (69.6)** |
| Cigarette smoking: n (%) | Never | 683 (28.2) | 1733 (71.8)** | 2258 (45.6) | 2693 (54.4) | 2941 (40.0) | 4426 (60.0)** |
|  | Past | 257 (43.3) | 337 (56.7) | 4 (36.3) | 7 (63.6) | 261 (43.1) | 344 (56.8) |
|  | Current | 268 (21.2) | 998 (78.8) | 12 (46.1) | 14 (53.9) | 280 (21.7) | 1012 (78.3) |
| Cigarette pack years: n (%) | =<10 | 198 (33.3) | 395 (66.6)** | 8 (44.4) | 10 (55.5) | 206 (33.7) | 405 (66.2)** |
|  | 11-28 | 168 (26.3) | 469 (73.6) | 1 (25.0) | 3 (75.0) | 169 (26.3) | 472 (73.6) |
|  | >28 | 156 (20.0) | 466 (74.9) | 1 (33.3) | 2 (66.6) | 157 (25.1) | 468 (74.8) |
| BMI (kg/m2): n (%) | <18 | 65 (23.6) | 210 (76.3) | 50 (43.1) | 66 (56.9) | 115 (29.4) | 276 (70.5) |
|  | 18-24.9 | 613 (28.2) | 1560 (71.7) | 687 (44.2) | 866 (55.7) | 1300 (34.8) | 2426 (65.1) |
|  | 25-29.9 | 406 (28.4) | 1023 (71.5) | 927 (45.8) | 1094 (54.1) | 1333 (38.6) | 2117 (61.3) |
|  | >=30 | 124 (31.0) | 275 (68.9) | 610 (47.0) | 688 (53.0) | 734 (43.2) | 963 (56.7)** |
| WC (cm) |  | 89.0 (11.8) | 88.7 (11.7) | 93.8 (12.5) | 92.4 (12.6)** | 92.2 (12.5) | 90.4 (12.3)** |
| *High WC: n (%)* |  | 422 (28.9) | 1035 (71.0) | 1980 (46.4) | 2283 (53.5)** | 2402 (41.9) | 3318 (58.0)** |
| SBP (mmHg) |  | 112.2 (18.6) | 111.2 (17.6) | 112.2 (20.3) | 112.5 (19.6) | 112.2 (19.7) | 111.8 (18.5) |
| DBP (mmHg) |  | 74.2 (11.74) | 73.4 (11.83) | 73.0 (12.21) | 73.4 (11.96) | 73.4 (12.06) | 73.4 (11.89) |
| *High BP: n (%)* |  | 373 (31.7) | 802 (68.2)** | 837 (46.4) | 966 (53.5) | 1210 (40.6) | 1768 (59.3)** |
| FBS (mg/dL) |  | 102.6 (29.8) | 103.3 (32.3) | 109.3 (43.5) | 106.2(37.6)** | 107.0 (39.4) | 104.6(34.9)** |
| *High FBS: n (%)* |  | 464 (26.3) | 1296 (73.6)* | 1043 (46.9) | 1178 (53.0) | 1507 (37.8) | 2474 (62.1) |
| HDL (mg/dL) |  | 54.4 (11.5) | 54.2 (11.9) | 59.5 (12.9) | 61.5 (13.3)** | 57.7 (12.7) | 57.6 (13.1) |
| *Low HDL: n (%)* |  | 156 (28.6) | 388 (71.3) | 648 (50.5) | 635 (49.4)** | 804 (44.0) | 1023 (55.9)** |
| Triglyceride (mg/dL) |  | 152.4 (106.8) | 155.1 (117.3) | 159.7 (103.2) | 151.8(95.0)** | 157.2 (104.5) | 153.6 (107.4) |
| *High TG: n (%)* |  | 449 (27.5) | 1182 (72.4) | 974 (47.7) | 1064 (52.2)** | 1423 (38.7) | 2246 (61.2) |
| Cholesterol (mg/dL) |  | 186.6 (40.0) | 187.6 (39.6) | 201.8 (41.8) | 203.7 (43.2) | 196.5 (41.8) | 195.2 (42.1) |
| LDL (mg/dL) |  | 101.9 (32.4) | 102.5 (32.1) | 110.3 (34.2) | 112.0 (35.6) | 107.3 (33.9) | 107.0 (34.1) |
| BMI: body mass index, WC: waist circumference, SBP: systolic blood pressure, DBP: diastolic blood pressure, BP: blood pressure, FBS: fasting blood sugar, HDL: high-density lipoprotein, TG: triglyceride, LDL: low-density lipoprotein  **p<0.05 **p<0.01*  Figures age, WC, SBP, DBP, FBS, HDL, TG, Total cholesterol, and LDL show mean (standard deviation). | | | | | | | |

**Supplementary Table S3. Baseline characteristics of the Pars cohort study population by waterpipe use and sex (Prevalence and Incidence of Metabolic Syndrome and Its Components among Waterpipe Users, Iran, 2024)**

**Supplementary Table S4. Waterpipe use and metabolic syndrome in the Pars cohort study, sensitivity analysis excluding individuals with chronic diseases (Prevalence and Incidence of Metabolic Syndrome and Its Components among Waterpipe Users, Iran, 2024)**

|  |  | **Number** | **Men**  **OR (95%CI)** | **Women**  **OR (95%CI)** | **Total**  **OR (95%CI)** |
| --- | --- | --- | --- | --- | --- |
| WP use excluding chronic diseases ^a^ | Never | 4897 | Reference | Reference | Reference |
|  | Past | 1075 | 1.49* (1.06 - 2.09) | 1.25* (1.00 - 1.56) | 1.33** (1.11 - 1.60) |
|  | Current | 1687 | 1.12 (0.83 - 1.52) | 1.23* (1.02 - 1.48) | 1.21* (1.04 - 1.42) |
| WP use excluding chronic diseases and cigarette smoking ^b^ | Never | 3765 | Reference | Reference | Reference |
|  | Past | 833 | 1.70* (1.04 - 2.78) | 1.22 (0.97 - 1.52) | 1.30* (1.06 - 1.58) |
|  | Current | 1487 | 1.07 (0.72 - 1.57) | 1.21 (1.00 - 1.46) | 1.19* (1.00 - 1.40) |
| OR: odds ratio, CI: confidence interval, PCS: pars cohort study, WP: waterpipe,  **p<0.05 **p<0.01*  ^a^ Subjects with a history of cardiovascular diseases, cerebrovascular accident, chronic obstructive pulmonary disease, chronic renal failure, jaundice and liver disease were excluded from the model.  ^b^ Subjects with a history of cardiovascular diseases, cerebrovascular accident, chronic obstructive pulmonary disease, chronic renal failure, jaundice and liver disease, and cigarette smoking were excluded from the model. | | | | | |

**Figure S1- Directed acyclic graph demonstrating the relationships between waterpipe use, metabolic syndrome (MetS), and potential covariates. (Prevalence and Incidence of Metabolic Syndrome and Its Components among Waterpipe Users, Iran, 2024)**


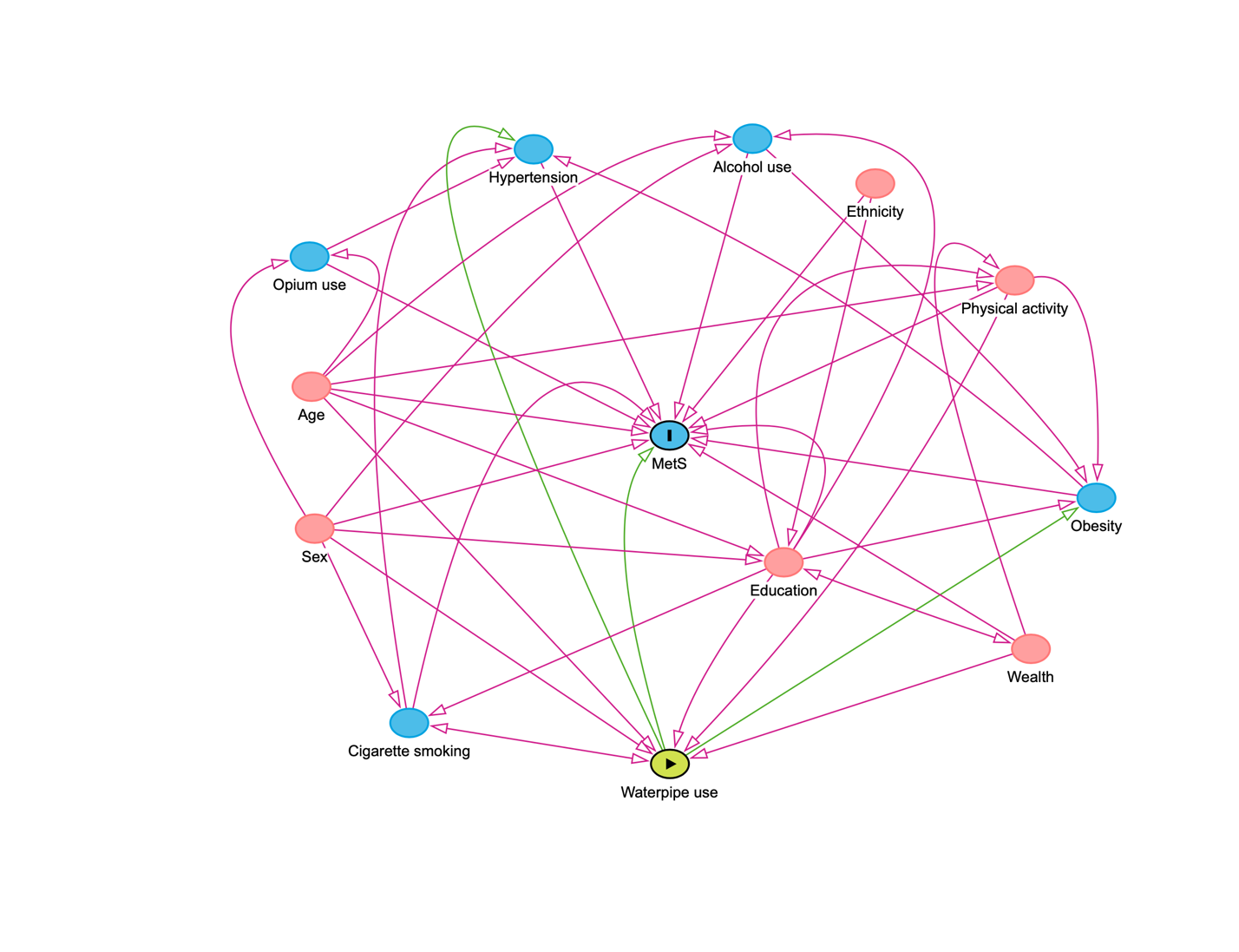

Supplement: Supplementary file 1 [file DataSheet1.docx]
